# The Role of Diet and Lifestyle in the Tinnitus Management: A Comprehensive Review

**DOI:** 10.7759/cureus.59344

**Published:** 2024-04-30

**Authors:** Smriti Wadhwa, Shraddha Jain, Nimisha Patil

**Affiliations:** 1 Otolaryngology - Head and Neck Surgery, Jawaharlal Nehru Medical College, Datta Meghe Institute of Higher Education and Research, Wardha, IND

**Keywords:** healthcare professionals, personalized approaches, management, lifestyle, diet, tinnitus

## Abstract

Tinnitus, the perception of noise or ringing in the ears without an external source, affects a significant portion of the global population. While there is no definitive cure, emerging research suggests that diet and lifestyle factors may play a role in tinnitus management. This comprehensive review explores the relationship between diet, lifestyle, and tinnitus, examining the existing evidence and potential mechanisms. The key findings highlight the influence of dietary patterns, hydration, stress management, physical activity, and sleep hygiene on tinnitus severity and frequency. Personalized approaches to tinnitus management are emphasized, recognizing the diverse nature of tinnitus symptoms and individual responses to interventions. Healthcare professionals are encouraged to integrate discussions about diet and lifestyle into tinnitus management protocols, while individuals affected by tinnitus are urged to adopt healthy habits and actively participate in their care. By addressing the multifaceted nature of tinnitus, healthcare professionals and individuals can collaborate toward optimizing symptom management and enhancing overall well-being.

## Introduction and background

Tinnitus, characterized by the perception of noise or ringing in the ears without an external sound source, affects a significant portion of the global population. Its manifestations vary widely, from occasional buzzing to constant ringing, impacting various aspects of daily life such as concentration and sleep quality [1]. Studies estimate that about 10-15% of people worldwide experience some degrees of tinnitus with 1-2% enduring severe and persistent symptoms [2]. The management of tinnitus poses a considerable challenge as there is no definitive cure. However, recent attention has been directed toward the influence of diet and lifestyle on tinnitus severity and frequency [3]. This emerging field suggests that certain dietary habits and lifestyle choices might play a role in either exacerbating or mitigating tinnitus symptoms. The recognition of the potential impact of these factors is vital for developing holistic approaches to address tinnitus effectively [4].

This comprehensive review aims to delve into the intricate relationship between diet, lifestyle, and tinnitus. By examining existing evidence and potential mechanisms, it seeks to shed light on how dietary patterns and lifestyle choices may influence tinnitus onset and severity. Furthermore, the review will provide practical recommendations for healthcare professionals and individuals affected by tinnitus, offering insights into dietary modifications, lifestyle adjustments, and other supportive strategies. In addition to synthesizing current knowledge, this review will also identify research gaps and propose avenues for future investigation. By pinpointing areas that require further exploration, it aims to contribute to a deeper understanding of the role of diet and lifestyle in tinnitus management. Through these efforts, we aspire to enhance the effectiveness of interventions and improve the quality of life for individuals grappling with this pervasive condition.

## Review

Understanding tinnitus

Causes and Mechanisms of Tinnitus

Tinnitus presents as a multifaceted symptom with diverse potential causes and mechanisms. It can stem from cochlear dysfunction, such as impairment to the outer hair cells in the inner ear, resulting in aberrant neural synchrony and alterations in tonotopic representation [5,6]. However, tinnitus can also emanate from various relevant anatomical structures along the central auditory pathways, with ample evidence indicating that many tinnitus forms arise from intricate interactions between peripheral and central mechanisms [5]. Beyond cochlear dysfunction, various factors may induce tinnitus, including exposure to loud noises, hearing impairment, emotional distress, and somatosensory influences [5]. Conceptually, tinnitus can be viewed as a pathology rooted in neural plasticity, with both molecular and systemic components. It encompasses a cochlear component linked to its onset phase and a central aspect tied to its long-term maintenance [5]. Insights from animal models of tinnitus have been instrumental in identifying the location and characteristics of underlying defects, revealing heightened spontaneous activity in regions like the dorsal cochlear nucleus, inferior colliculus, and primary auditory cortex, alongside increased neural synchrony and bursting activity along the auditory pathway [6]. Spontaneous otoacoustic emissions (SOAEs) represent one potential source of tinnitus, termed cochlear mechanical tinnitus. They are typically mild occurrences, often encountered in individuals with normal hearing and those affected by middle-ear ailments [7]. Nonetheless, tinnitus also emerges due to neuroplastic changes within the central auditory pathway and somatosensory alterations, complicating the elucidation of its exact subjective mechanism [7].

Types of Tinnitus

Tinnitus encompasses various types, with subjective and objective tinnitus being the primary classifications. Subjective tinnitus comprises approximately 99% of cases and is audible solely to the individual experiencing it [8,9]. It can arise from diverse factors such as sudden exposure to loud noises, aging, hearing impairment, or conditions like Ménière's disease [9]. In contrast, objective tinnitus, a rare occurrence affecting only about 1% of tinnitus sufferers, can be heard by the affected person and others, including medical professionals employing a stethoscope [9]. Typically, objective tinnitus stems from vascular anomalies, neurological disorders affecting facial muscles, or patulous eustachian tubes, a condition characterized by the persistent opening of these tubes [9]. Additional varieties of tinnitus include somatic tinnitus, associated with movements or tactile sensations, often triggered by muscle spasms, neck misalignments, or dental issues [10]. Pulsatile tinnitus, exhibiting a rhythmic pattern synchronized with the heart's beats, indicates alterations in blood flow near the ear [10]. Low-frequency tinnitus, affecting individuals profoundly with tones corresponding to the lowest octaves on a piano, manifests as humming, murmuring, rumbling, or deep droning sounds [10]. Musical tinnitus, also known as musical hallucinations or auditory imagery, is a less common type characterized by the perception of simple tones or layered melodies, prevalent among individuals with prolonged hearing loss and tinnitus history [10]. Treatment strategies for tinnitus vary depending on its type and underlying cause. Behavioral interventions and sound-generating devices yield favorable outcomes, while hearing aids can alleviate symptoms in individuals with hearing impairment [10].

Impact of Tinnitus on Quality of Life

Tinnitus profoundly impacts individuals' quality of life, affecting diverse facets such as psychological well-being, emotional stability, sleep patterns, auditory functions, and overall health-related quality of life (HRQoL) [11]. Its presence often correlates with lower HRQoL due to associated factors like hearing loss, ototoxicity, head injury, and depression, presenting a formidable challenge to individuals' overall well-being [12]. Studies underscore the emotional toll of tinnitus, revealing a spectrum of reactions including difficulty concentrating, frustration, anger, and even depression, all of which significantly disrupt daily functioning and emotional equilibrium [13,14]. The adverse effects of tinnitus extend beyond the auditory realm, encompassing psychological and emotional distress, sleep disturbances, and broader health repercussions [13,14]. Moreover, tinnitus can prove debilitating, impeding individuals' ability to focus, sleep, and participate in routine activities, consequently diminishing their overall quality of life [13,15]. Furthermore, tinnitus often triggers additional complaints beyond the perceptual aspect of sound, manifesting as heightened stress levels, concentration difficulties, and disrupted sleep patterns, all of which collectively contribute to a diminished HRQoL among those grappling with tinnitus [12].

Diet and tinnitus

Nutritional Factors Influencing Tinnitus

Micronutrients (vitamins and minerals): Micronutrients, comprising vitamins and minerals, are indispensable for the body despite being needed in minute quantities [16,17]. They fulfill various functions, facilitating enzyme and hormone production essential for normal growth and development [17]. Water-soluble vitamins, such as vitamins B and C, dissolve in water and necessitate daily replenishment as they are not stored in the body [18]. In contrast, fat-soluble vitamins like A, D, E, and K dissolve in fat and can be stored for later use [18]. Microminerals such as calcium, magnesium, sodium, and potassium are crucial for muscle and bone health and contribute to blood pressure regulation [18]. Trace minerals like iron, manganese, copper, zinc, and selenium play pivotal roles in muscle health, nervous system function, and cell repair [18]. Most micronutrients are sourced from food, emphasizing the importance of a varied diet [18].

Macronutrients (fats, proteins, and carbohydrates): Carbohydrates serve as the primary energy source for the body, vital for various physiological functions including central nervous system, brain, kidney, and muscle function [19]. They can be stored for later use and play a key role in intestinal health and waste elimination. Carbohydrate sources encompass simple sugars in honey and fruits and complex starches in grains, potatoes, and starchy vegetables [19]. Proteins, essential for growth, tissue repair, and maintaining lean body mass, are composed of amino acids, some of which must be acquired through diet [20]. Protein-rich foods include animal products like meat, poultry, fish, dairy, and plant-based sources like beans, lentils, nuts, seeds, and soy [20]. Fats are vital for energy storage, organ cushioning, hormone synthesis, vitamin absorption, and cell membrane integrity [21]. While trans fats should be limited, unsaturated fats in olive oil, avocados, and nuts benefit heart health. Fats are the most calorie-dense macronutrient, providing over twice the calories per gram compared to proteins and carbohydrates [21].

Dietary patterns and tinnitus: Research on dietary patterns and tinnitus aims to uncover potential relationships between them. While evidence linking specific foods or drinks to tinnitus severity remains inconsistent, some studies suggest dietary factors may influence conditions like Ménière’s disease, affecting the inner ear [22]. However, conclusive links are lacking for other types of tinnitus, with research presenting contradictory findings [22]. For instance, a 2018 study found associations between persistent tinnitus and higher intake of fruits, vegetables, bread, fish, and eggs, while dairy and caffeinated coffee intake were linked to reduced odds of persistent tinnitus [4]. Additionally, higher caffeine intake was associated with a lower risk of incident tinnitus in women [23]. Similarly, a healthier diet, as indicated by a higher Healthy Eating Index (HEI) score, was correlated with reduced odds of reported persistent tinnitus in cross-sectional analysis [24].

Role of Hydration in Tinnitus Management

Hydration is pivotal in managing tinnitus, as dehydration can worsen symptoms and contribute to conditions that trigger tinnitus, such as high blood pressure, compromised hearing health, and ear infections [25]. Ensuring adequate hydration is essential for sustaining healthy hearing, as the ears rely on fluid for optimal function [25]. Studies have indicated that decreased vitamins B2, B3, water, and protein intake may correlate with tinnitus and related discomfort [26]. Moreover, research conducted in Korea revealed a significant association between water intake and tinnitus, with individuals experiencing tinnitus displaying lower water consumption [26]. This finding underscores the significance of maintaining adequate hydration, particularly during middle age when many individuals are engaged in active careers [26]. Given the impact of hydration on tinnitus management, clinicians should advise patients to maintain a healthy body weight and ensure sufficient intake of essential nutrients, including vitamins B2, B3, water, and protein [26].

Potential Dietary Triggers of Tinnitus

Potential dietary triggers of tinnitus encompass caffeine, sodium, salicylates, aspartame, sugar, and unhealthy fats. Caffeine, prevalent in beverages like coffee, tea, hot chocolate, and energy drinks, can exacerbate tinnitus symptoms by elevating blood pressure and stimulating nerve cell activity [27]. Sodium, commonly found in processed and fast foods and snack items, can constrict blood vessels and elevate blood pressure, worsening tinnitus symptoms [27]. Salicylates, natural compounds in fruits, vegetables, nuts, and oils, may accumulate in the body and provoke adverse reactions, potentially exacerbating tinnitus symptoms in sensitive individuals [27]. Aspartame, an artificial sweetener, is suspected to be linked to tinnitus due to its potential toxicity to the brain and inner ear, particularly under conditions of heat exposure or prolonged storage [27]. Problems with sugar metabolism, such as hyperinsulinemia, may also contribute to tinnitus symptoms, and adherence to a diabetic diet could alleviate symptoms for some individuals [4]. Unhealthy fats, including saturated and trans fats, can impede circulation and diminish blood flow, potentially intensifying tinnitus severity [27]. Consequently, individuals experiencing tinnitus may find it beneficial to monitor their dietary habits and make appropriate adjustments to avoid potential triggers.

Lifestyle factors and tinnitus

Stress Management Techniques and Their Effect on Tinnitus

Effective stress management techniques can significantly ameliorate tinnitus symptoms and alleviate the associated distress. Tinnitus and stress share a close relationship, with stress often exacerbating tinnitus symptoms, while the condition itself can induce stress and anxiety [28,29]. The research underscores stress as a trigger for tinnitus or a factor in worsening existing symptoms, creating a cyclic interplay between stress and tinnitus [29]. An array of stress management strategies can be implemented to disrupt this cycle. Relaxation techniques, such as deep breathing exercises, visualization, and self-hypnosis, have effectively reduced stress levels and tinnitus symptoms [28]. Mindfulness-based tinnitus stress reduction programs, which emphasize deep breathing, yoga, relaxation, and meditation, offer promise in tinnitus management [30]. Additionally, mitigating stress through exercise, problem-solving, engaging in enjoyable activities, and socializing can contribute to tinnitus management [31]. Activities that reduce overall stress levels may positively impact tinnitus perception and reactions, given the interconnectedness of stress and tinnitus [30]. Cognitive-behavioral therapy (CBT) serves as another valuable tool in tinnitus management by assisting individuals in identifying and reframing negative thoughts regarding tinnitus into more constructive ones [30]. Often utilized alongside mindfulness programs, CBT has demonstrated efficacy in reducing depression and anxiety, enhancing social functioning, and improving overall mental well-being [30]. Furthermore, adopting a daily exercise regimen, learning to regulate stress responses, and seeking support from healthcare professionals or tinnitus support groups are valuable components of stress and tinnitus management [29]. Soft music, white noise, specialized tinnitus relaxers, and apps that generate relaxing sounds can effectively distract from tinnitus symptoms [29,30].

Impact of Physical Activity and Exercise on Tinnitus

The influence of physical activity and exercise on tinnitus is a multifaceted issue characterized by both positive and negative ramifications. On one hand, regular physical activity has demonstrated notable benefits in enhancing health-related and overall quality of life while concurrently diminishing levels of tinnitus distress [32]. A study revealed a significant association between higher physical activity levels and improved health-related and global quality of life, coupled with reduced tinnitus distress [32]. Furthermore, physical activity emerged as a significant factor contributing to variations in tinnitus severity, indicating its potential as a management strategy for affected individuals [32]. Conversely, certain forms of exercise can precipitate or exacerbate tinnitus symptoms. High-impact aerobics, such as running, basketball, football, and soccer, can potentially dislodge otoconia and calcium crystals within the ears, leading to inner ear issues and tinnitus exacerbations [33]. Additionally, exerting strain while lifting weights or engaging in heavy exertions can elevate intracranial pressure, exerting pressure on the ears and potentially resulting in perilymph fistula, which is a tear in the membrane separating the middle and inner ear, manifesting as dizziness, tinnitus, and heightened hearing sensitivity [33]. Hence, it is imperative to recognize the potential impact of exercise on tinnitus and seek medical guidance if experiencing ringing or buzzing in the ears following physical activity. It is advisable to incorporate adequate rest days, engage in proper warm-up routines, and vary physical activities to mitigate the risk of exercise-induced tinnitus [33]. Moreover, certain exercises such as yoga, tai chi, gentle swimming, meditation, acupuncture, massage therapy, and sexual activity can stimulate the release of endorphins, which possess analgesic properties and promote stress reduction, potentially benefiting individuals with tinnitus [34].

Sleep Hygiene and Its Relationship With Tinnitus

Maintaining proper sleep hygiene is crucial for effectively managing tinnitus symptoms, especially for individuals experiencing difficulties falling asleep, frequent awakenings, or poor sleep quality due to tinnitus. A systematic review of studies focusing on lifestyle-related risk factors for tinnitus revealed that smoking significantly heightens the risk of developing tinnitus. At the same time, findings regarding alcohol consumption and caffeine intake or coffee consumption are mixed [35]. Sleep disturbances are prevalent among individuals with tinnitus, as silence can often accentuate tinnitus symptoms at bedtime. To mitigate tinnitus during nighttime, experts recommend employing white noise or other soothing sounds to mask the tinnitus sound, elevating the pillow to alleviate congestion, and establishing a consistent bedtime routine that incorporates stress-reducing activities [36]. Reducing the consumption of caffeine, alcohol, and tobacco is imperative for managing tinnitus symptoms, as these substances can exacerbate tinnitus. Additionally, consulting a healthcare professional about new, persistent, or bothersome tinnitus can aid in identifying the underlying cause and receiving appropriate treatment recommendations [36]. CBT offers counseling to help individuals shift their responses to tinnitus symptoms. In contrast, tinnitus retraining therapy (TRT) aims to modify the brain's response to tinnitus, rendering the sounds less noticeable and bothersome. Biofeedback is another technique that assists individuals in learning to regulate their body's physical response to tinnitus symptoms [36].

Substance Use (Caffeine, Alcohol, Nicotine) and Tinnitus

Substance use, encompassing caffeine, alcohol, and nicotine, has been linked to tinnitus. A comprehensive review of 384 studies revealed that both current and past smokers exhibited a significantly heightened risk of tinnitus, whereas alcohol consumption did not yield a notable effect. However, the review did not identify a significant impact of caffeine intake or coffee consumption on tinnitus risk. Notably, rock musicians, frequently exposed to loud noise, exhibit an elevated prevalence of tinnitus and anxiety. Moreover, chronic tinnitus correlates with depressive symptoms among rock musicians, with tinnitus-affected musicians reporting depressive symptoms significantly more often than controls (13.6% vs. 5%) [37]. Drug-induced tinnitus and other hearing disorders have been associated with a broad spectrum of drugs, encompassing both acute intoxication and long-term medication use [38]. It's essential to recognize that while certain medications may offer relief for patients grappling with significant behavioral issues, there is scant scientific evidence supporting their efficacy in measurably improving tinnitus [39]. Over-the-counter substances marketed as "tinnitus remedies" or "miracle cures" lack scientifically measurable effects and should be approached with caution [39].

Clinical evidence and recommendations

Studies Investigating the Relationship Between Diet/Lifestyle and Tinnitus

Multiple studies have explored the nexus between diet and lifestyle factors and tinnitus. A UK study involving over 34,000 adults revealed that elevated calcium, iron, and fat intakes were linked to heightened tinnitus odds. In contrast, increased intakes of vitamin B12 and adherence to a dietary pattern rich in meat intake were associated with reduced odds of tinnitus [40]. This study underscored the potential influence of nutrients on the onset of tinnitus. In Italy, a hospital-based case-control study comprising 185 incident idiopathic tinnitus cases unveiled that moderate-to-high consumption of caffeine, butter, poultry, prosciutto, and legumes exhibited an inverse association with tinnitus onset. At the same time, dietary variety mitigated the risk of tinnitus [41]. These findings underscored the significance of dietary factors in tinnitus onset and suggested a potential inverse correlation between protein-rich foods and tinnitus. Furthermore, the Mediterranean diet has emerged as a recommended dietary approach for individuals grappling with tinnitus, advocating daily physical activity and a diet abundant in whole grains, fruits, vegetables, olive oil, fish, poultry, eggs, and limited red meat consumption [42]. This dietary regimen promotes overall health and holds promise in alleviating tinnitus symptoms. Collectively, these studies offer valuable insights into the interplay of diet and lifestyle with tinnitus, suggesting that specific nutrients and dietary patterns may influence the onset and severity of tinnitus. Nonetheless, further research is warranted to validate these findings and formulate tailored dietary recommendations for effective tinnitus management.

Practical Recommendations for Dietary and Lifestyle Modifications in Tinnitus Management

**Figure 1:**
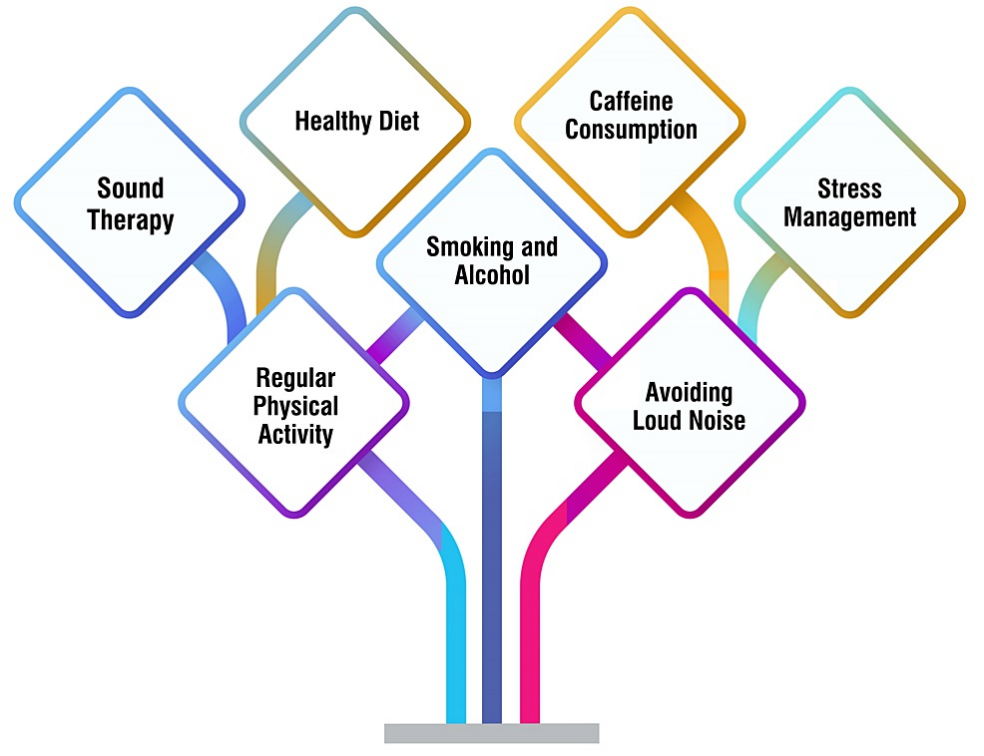
Practical recommendations for dietary and lifestyle modifications in tinnitus management Image credit: Dr Smriti Wadhwa

Adopting a health-conscious diet is paramount for reducing hypertension, enhancing blood flow, boosting energy levels, and fostering emotional well-being, all of which can positively influence tinnitus symptoms [43]. Particularly for patients with tinnitus and Ménière’s disease, exploring a low-salt diet is crucial, given the strong correlation between salt consumption and Ménière’s symptoms [43]. While scientific evidence on caffeine exacerbating tinnitus symptoms remains limited, patients should monitor their responses to caffeine and adjust consumption accordingly. If caffeine appears to worsen tinnitus, reducing intake may prove beneficial [43]. Effective stress management plays a pivotal role in minimizing the impact of tinnitus symptoms. Regular physical activity and exercise can lower stress levels, alleviating tinnitus symptoms [44]. Furthermore, involvement in social activities, recreational hobbies, and sharing experiences with others can distract from tinnitus, fostering emotional well-being [43]. Limiting exposure to loud noises is crucial for preventing further hearing damage and potentially exacerbating tinnitus symptoms. Whether in the workplace, at concerts, or during personal activities, taking precautions to minimize exposure to loud noises is essential [43]. Quitting smoking and reducing alcohol consumption can significantly enhance overall bodily health and potentially decrease the likelihood of tinnitus flare-ups [45]. Regular physical activity maintains physical fitness and plays a pivotal role in managing tinnitus symptoms by reducing stress levels [44]. Sound therapy, such as white noise machines or masking devices, can effectively mask tinnitus and relieve some individuals [46]. Incorporating sound therapy into daily routines can help alleviate the distress associated with tinnitus. Practical recommendations for dietary and lifestyle modifications in tinnitus management are shown in Figure 1.

Challenges and Limitations in Implementing Dietary and Lifestyle Changes

Implementing dietary and lifestyle changes can present challenges due to various individual, social, and environmental factors. Common obstacles to adopting healthy lifestyle practices include a lack of self-motivation, busy schedules, time constraints, consumption of high-calorie beverages and fast food, indulgence in high-calorie snacks and fatty foods, rapid eating habits, meal skipping, frequent consumption of fried or breaded foods, late-night eating, excessive use of high-fat or calorie-laden condiments and salad dressings, oversized portion sizes, frequent indulgence in calorie-rich desserts, influence from significant others, home environment factors, tendency to make excuses, stress, and environmental and physical barriers to exercise [47]. For families, the considerable time investment required for planning, purchasing ingredients, meal preparation, cooking, and cleanup can pose a substantial hindrance, particularly for families with demanding work schedules or where only one parent is available to manage meal-related tasks [48]. Furthermore, the stress associated with implementing healthy lifestyle changes can lead to tension and overwhelm within the household [48]. Additionally, altering dietary habits can be particularly challenging given the essential role of food in daily life, unlike habits such as smoking or drinking, which can be more easily modified [48]. To surmount these barriers, it is essential to identify individual impediments to lifestyle changes and devise strategies to overcome them. This may entail generating realistic solutions through brainstorming, evaluating the advantages and disadvantages of each solution, and selecting the most suitable approach to facilitate desired lifestyle modifications [47]. For healthcare providers, addressing barriers to dietary and lifestyle changes is vital for achieving successful patient outcomes. This may involve furnishing patients with education and resources to assist them in overcoming obstacles, such as offering guidance on meal planning and preparation, imparting stress management techniques, and providing recommendations for physical activity [49].

Future directions and research needs

Areas for Further Research on Diet and Lifestyle Interventions for Tinnitus

**Figure 2:**
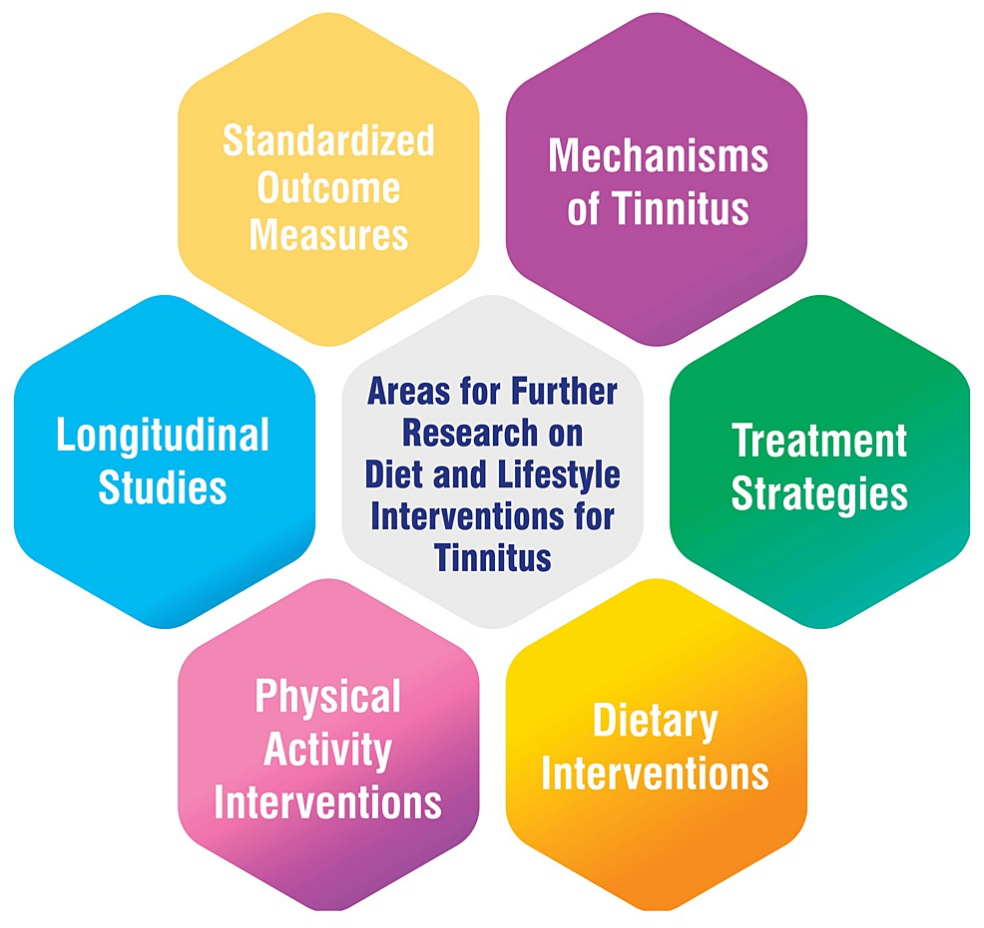
Areas for further research on diet and lifestyle interventions for tinnitus Image credit: Dr Smriti Wadhwa

Tinnitus research focuses on several key areas to enhance understanding and treatment effectiveness. One crucial aspect involves developing standardized outcome measures and establishing consensus on relevant outcome domains for tinnitus trials, aiming to elevate the quality and comparability of future studies [50]. Additionally, there is a growing interest in delving deeper into the diverse symptoms and underlying mechanisms of tinnitus, extending beyond the conventional notion of "ringing in the ears." This expanded exploration seeks a more comprehensive understanding of tinnitus symptoms, potentially leading to more tailored and effective treatment strategies [22]. Exploration of treatment strategies remains a priority, with ongoing investigation into modalities such as sound therapy, CBT, and transcranial magnetic stimulation (TMS). The aim is to elucidate their long-term safety, efficacy, and potential for clinical application in managing tinnitus [50]. Moreover, research is underway to assess the impact of specific dietary interventions on tinnitus symptoms. Factors under consideration include carbohydrate and lipid metabolism, the role of protein-rich foods, and the influence of weight loss on tinnitus severity and onset [51,52]. Another area of interest is understanding the effects of physical activity interventions, either alone or combined with dietary changes, on tinnitus symptoms and quality of life. This line of inquiry, particularly relevant for individuals with tinnitus and obesity, aims to shed light on potential lifestyle interventions to alleviate tinnitus symptoms [52]. Furthermore, longitudinal studies are being conducted to validate associations between dietary habits, lifestyle factors, and tinnitus onset. Such studies aim to provide more robust evidence for developing dietary recommendations tailored to tinnitus management [41]. Areas for further research on diet and lifestyle interventions for tinnitus are shown in Figure 2.

Emerging Trends in Tinnitus Management

Emerging trends in tinnitus management encompass various innovative approaches to understanding and alleviating this condition. One notable trend involves utilizing animal models and advanced electrophysiological recording techniques to delve into the neural basis of tinnitus. Additionally, researchers are exploring somatic tinnitus, wherein movements of the eyes, head, neck, jaw, and shoulder can influence tinnitus loudness and pitch. Treatments such as low-frequency repetitive transcranial magnetic stimulation (rTMS) and sequential therapies are being investigated in this regard [53]. In addressing chronic subjective tinnitus, current and emerging therapies encompass a diverse array of treatments, including CBT, TRT, sound therapy, TMS, and transcutaneous electrical stimulation. Of particular interest is the adoption of bimodal stimulation approaches, integrating multisensory modalities, which have garnered attention for their potential efficacy in tinnitus management [54]. Recent updates have shed light on the efficacy of certain drugs for chronic tinnitus, with findings indicating the ineffectiveness of substances like Ginkgo biloba extract, St. John's wort, antidepressants, benzodiazepines, zinc, melatonin, cannabis, oxytocin, steroids, and gabapentin. However, combining Ginkgo biloba extract and hyaluronic acid has significantly improved tinnitus severity, offering a promising avenue for further exploration [3].

Potential Integration of Technology in Dietary and Lifestyle Interventions for Tinnitus

Integrating technology into dietary and lifestyle interventions for tinnitus management represents a significant advancement in treatment approaches. Widex Zen Therapy (WZT) is a well-documented option, combining sound therapy with counseling to effectively address tinnitus symptoms [55]. Another emerging trend involves using fractal music, which has shown promise in improving functional scores among chronic tinnitus patients, as evidenced by improvements in the tinnitus handicap inventory [56]. Furthermore, a system medicine approach has been proposed to tackle comorbid conditions as manifestations of a disrupted system and to develop early risk predictors for chronification, a strategy applicable to tinnitus management [56]. This approach integrates traditional medicine with modern technologies, including mobile health (mHealth) applications, to deliver more precise, personalized recommendations for managing chronic tinnitus through dietary interventions. Despite progress, identifying individuals who may benefit from dietary modifications remains challenging. A large-scale online survey revealed that while certain dietary items like caffeine, alcohol, and salt were likely to influence tinnitus severity, their impact was significant for only a small proportion of participants [56]. Therefore, ongoing efforts are needed to enhance the clinical utility of identifying those who may benefit from dietary changes. Incorporating foods rich in antioxidants and omega-3 fatty acids has positively affected tinnitus symptoms. Additionally, the advent of mobile apps and wearables has ushered in a new era of self-management for individuals with tinnitus [41]. These innovative tools offer many features, including sound therapy programs, relaxation exercises, and educational modules, empowering individuals with tinnitus with valuable resources and strategies to manage their symptoms effectively.

## Conclusions

In conclusion, this review underscores the nuanced relationship between diet, lifestyle, and the management of tinnitus. Through exploration, uncovered compelling evidence suggesting that specific dietary patterns and lifestyle choices may influence the severity and frequency of tinnitus symptoms. However, it's apparent that personalized approaches tailored to individual needs and circumstances are essential for optimizing outcomes. Healthcare professionals are encouraged to integrate discussions about diet and lifestyle into tinnitus management protocols, offering tailored guidance and support. Simultaneously, individuals affected by tinnitus are urged to actively engage in their care by adopting healthy dietary habits, managing stress levels, prioritizing sleep hygiene, and embracing other lifestyle modifications conducive to symptom alleviation. By fostering collaboration between healthcare professionals and individuals living with tinnitus, we can strive toward achieving better symptom management and ultimately enhancing overall quality of life.
